# Intelligent Brushing Monitoring Using a Smart Toothbrush with Recurrent Probabilistic Neural Network

**DOI:** 10.3390/s21041238

**Published:** 2021-02-10

**Authors:** Ching-Han Chen, Chien-Chun Wang, Yan-Zhen Chen

**Affiliations:** Machine Intelligence and Automation Technology Lab, Department of Computer Science & Information Engineering, National Central University, No. 300, Zhongda Rd., Zhongli Dist., Taoyuan City 320, Taiwan; pierre@g.ncu.edu.tw (C.-H.C.); 104582004@cc.ncu.edu.tw (Y.-Z.C.)

**Keywords:** smart toothbrush, Bass Brushing Technique, recurrent probabilistic neural network, posture recognition

## Abstract

Smart toothbrushes equipped with inertial sensors are emerging as high-tech oral health products in personalized health care. The real-time signal processing of nine-axis inertial sensing and toothbrush posture recognition requires high computational resources. This paper proposes a recurrent probabilistic neural network (RPNN) for toothbrush posture recognition that demonstrates the advantages of low computational resources as a requirement, along with high recognition accuracy and efficiency. The RPNN model is trained for toothbrush posture recognition and brushing position and then monitors the correctness and integrity of the Bass Brushing Technique. Compared to conventional deep learning models, the recognition accuracy of RPNN is 99.08% in our experiments, which is 16.2% higher than that of the Convolutional Neural Network (CNN) and 21.21% higher than the Long Short-Term Memory (LSTM) model. The model we used can greatly reduce the computing power of hardware devices, and thus, our system can be used directly on smartphones.

## 1. Introduction

The occurrence of chronic illnesses is a very common phenomenon in society, such as high blood pressure, diabetes, heart disease, etc., in adults and dental caries, periodontal disease and Gingivitis in both children and adults [1]. Most of these dental diseases are the result of bacterial deposition on the surfaces of teeth [2]. If a tooth is not brushed properly, then bacteria will accumulate on its surface, forming plaque, destroying the outermost layer of the tooth (enamel) and triggering gingivitis, which can lead to dental caries and gum disease. Thoroughly cleaning teeth can effectively reduce tooth plaque and prevent oral diseases.

Brushing one’s teeth every day is the primary method to prevent various oral diseases. The American Dental Association (ADA) and the Taiwan Dental Association both recommend using the Bass Brushing Technique to brush your teeth and to do it at least twice a day for two minutes [3,4]. However, studies have pointed out that most people’s brushing time is insufficient, and the average brushing time per person is between 30 s and 60 s [5,6,7,8]. For a brushing time of less than two minutes, each tooth’s brushing time is not enough [9,10]. The Bass Brushing Technique involves the following: (1) One must brush the outer side of the front row of teeth, the outer side of the left and right rows of teeth, and the inner side of the left and right rows of teeth. The toothbrush is at a 45-degree angle with the gums. These areas are brushed vertically with the toothbrush. Each tooth brushing starts from the gum line, starting with the tip of the tooth. (2) When brushing the chewing surface of the tooth, move the bristles of the toothbrush back and forth along the chewing surface of the tooth. (3) When brushing the inside of the front row of teeth, tilt the toothbrush vertically and move it up and down.

According to forecasts from UK market research organizations, more than 400 million smart wearable devices will be sold by 2020, with an estimated value of $34 billion [11]. Inertial sensors have been widely used in different wearable devices, such as smart watches, sports bracelets, smart sports shoes, smart glasses, Bluetooth headsets, etc., that are all embedded with Inertial Measurement Unit (IMU). It can detect the motion state of the device and identify various motion postures in different fields [12]. In the field of healthcare, inertial sensor data can be used to monitor the onset and recovery of diseases, such as assessing the recovery of patients during rehabilitation [13,14,15] and detecting and diagnosing diseases [16,17]. In the field of home care, a care system can provide monitoring or assistance to the occupants to ensure their health, safety, and good physical condition, such as a tracking and monitoring emergency help system [18] to assist patients with physical and mental disabilities [19,20,21]. In the field of sports and leisure, wearable sensor devices can be used to identify people’s sports and leisure activities and improve their quality of life, such as daily action recognition [22,23] and motion pose recognition [24,25].

In many of the above areas, the application of inertial sensors in the field of healthcare is particularly noteworthy. These studies show that there is broad development in the use of inertial sensors for human activity recognition (HAR) in healthcare applications. Space, the busy lives of people, and the increase of the elderly population make healthcare an urgent problem, and through the integration of digital technology, the healthcare industry can reduce the cost of care for humans and financial services and improve the quality of healthcare. Unlike these applications, toothbrushes are used to clean teeth. Brushing includes a series of movements for manipulating the toothbrush. The toothbrush and the user’s movements are closely combined. Therefore, the data extracted by the sensor needs to be used to accurately identify the user. For the operation of the toothbrush, even after learning the Bass Brushing Technique, there will be some differences in the brushing movements of different users. Due to the small space in the mouth, the range of the toothbrush can be small, and the classification of the brushing needs many regions, making it difficult to accurately classify small-scale actions. Establishing accurate models and effective monitoring is a very challenging topic.

On the topic of brushing motion recognition, studies have looked at how to use manual toothbrushes and smart watches to monitor the brushing quality of all tooth surfaces [26] and to capture brush movements and directions through a magnetometer attached to the toothbrush handle and a magnetic sensor in the watch. Based on the inertial sensing data from the watch, the brushing posture can be recognized, and the sound signal collected from the watch is used to assist the recognition. This method can be used with a smart watch, and the average recognition rate is only 85.6%. J.W. et al. [27] of Korea’s Konkuk University employed a three-axis accelerometer on the bottom of the toothbrush to measure the user’s brushing posture. This method only utilizes a three-axis accelerometer to measure motion information, and it cannot stably represent all brushing movements. This way is easy to cause misjudgment. In addition, research [28] took an accelerometer, magnetic sensor, and Euler angle as identification features and used the K-means algorithm to identify and classify 15 brushing areas but could not meet the correctness and integrity of brushing. Each research method has its own room for improvement.

The rise of deep learning in recent years has made deep learning widely applied in sequence learning-related research, such as image recognition [29,30,31], speech recognition [32,33], and sensor data analysis [34,35]. There are many studies that have begun to employ deep learning methods to sensor motion recognition [36]. Traditional human body recognition methods, such as the Hidden Markov Model (HMM) [37], specifically deal with the relationship between many random variables in the sequence data. The hidden Markov model uses the graph probability model to describe the problem, which has the Markov property. The current state is conditionally independent of the past state, and so, it can only learn the shallower features [38], resulting in a reduced effectiveness in unsupervised learning and limited classification accuracy and model promotion. Generally speaking, static data can only be used as a learning basis, and the features need to be extracted manually. However, in real life, motion data usually present a dynamic sequence, which requires stable and continuous learning.

Different from traditional human motion recognition methods, deep learning does not need to extract features manually. It can input unmarked data as features. Since the data collected by sensor data are time series, some studies have proposed using convolution. The Convolutional Neural Network (CNN) and the Long Short-Term Memory (LSTM) model are used to identify sensor data [39,40]. This method does not require preprocessing of the data. It uses raw data directly as a deep neural network input.

Although deep learning has many of the above advantages, its immediacy and computing resources are still not suitable for embedded systems. Deep learning in a neural network architecture refers to a neural network model with multiple hidden layers. There are many layers and nodes, which contain many parameters and require a large amount of training data to adjust the parameters. The model complexity is extremely high. To develop an effective deep learning model within a reasonable time, it is necessary to use the acceleration function of the Graphics Processing Unit (GPU). Therefore, the hardware cost required for typical deep learning is high, the computing resources are expensive, and it is not easy to apply to smart toothbrushes. Aiming at the shortcomings of existing smart toothbrushes, such as high cost, low recognition accuracy, and a lack of personal adaptation, this research plans an innovative deep neural network classifier that combines smart toothbrushes with nine-axis motion sensors and visualization software on a mobile phone, which can be applied to monitor the correctness and completeness of the brushing process for protecting oral health.

## 2. Formalization of Brushing Posture

**Figure 1:**
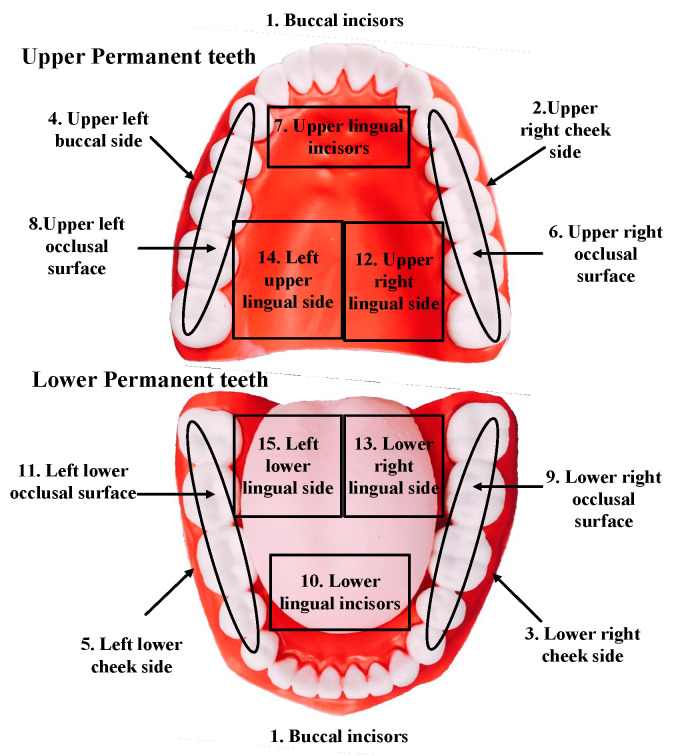
Fifteen brush cleaning areas.

This study is based on the Bass Brushing Technique’s brushing action guidelines, which divide the teeth into 15 brushing areas to describe the brushing area and posture. The fifteen brush cleaning areas are first defined, as shown in Figure 1.

**Figure 2:**
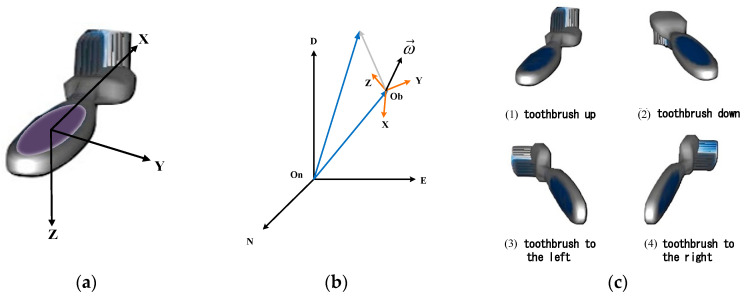
Brushing coordinates: (**a**) carrier coordinate system, (**b**) fixed coordinates and carrier coordinates, and (**c**) toothbrush steering.

In order to identify the brushing posture, we define the carrier coordinate frame of the toothbrush. The origin is located at the center of gravity of the carrier (toothbrush). The directions of the X, Y, and Z axes are usually located directly in the forward and lateral directions of the carrier and directly below the carrier. As shown in Figure 2a, the gravitational acceleration (G = 1 g) is used as the reference vector to calculate the attitude angle, and the gravitational acceleration direction is defined as the direction of the Z-axis coordinate. In rigid kinematics, the acceleration of a particle moving in space can be compared with a fixed coordinate system and a motion coordinate. Therefore, we used the North-East-Down Coordinate System (NED) as the fixed coordinate and the carrier coordinate (the body frame) as the moving coordinate (Figure 2b). The features used in the brushing area recognition are then defined as the angle of the toothbrush’s steering and brushing. We first use turning the toothbrush (Figure 2c) to classify the brushing posture and then use the attitude angle as the recognition area of the brushing area.

When we attach the nine-axis sensor to the toothbrush, we can measure the gravitational acceleration of the toothbrush. We use the gravitational acceleration measured by the toothbrush and the rotation angle of the Euler angle as the recognition features of the toothbrush steering. The elevation angle and the roll angle formula are as follows:

(1) $$Pitch=\rho =\sin^{-1}\left(A_{y}\right)$$

(2) $$Roll=\gamma =-\sin^{-1}\left(\frac{A_{x}}{\cos\rho }\right)$$

The brushing system takes the coordinates on the brush holder as the origin, and the sensor will automatically correct the sensor value of the toothbrush on the toothbrush holder to be close to zero each time the toothbrush is used. When the user grabs the toothbrush, the brushing is started in front of the toothbrush. From brushing gesture recognition, Table 1 defines the range of toothbrush steering characteristic values for this project.

**Figure 3:**
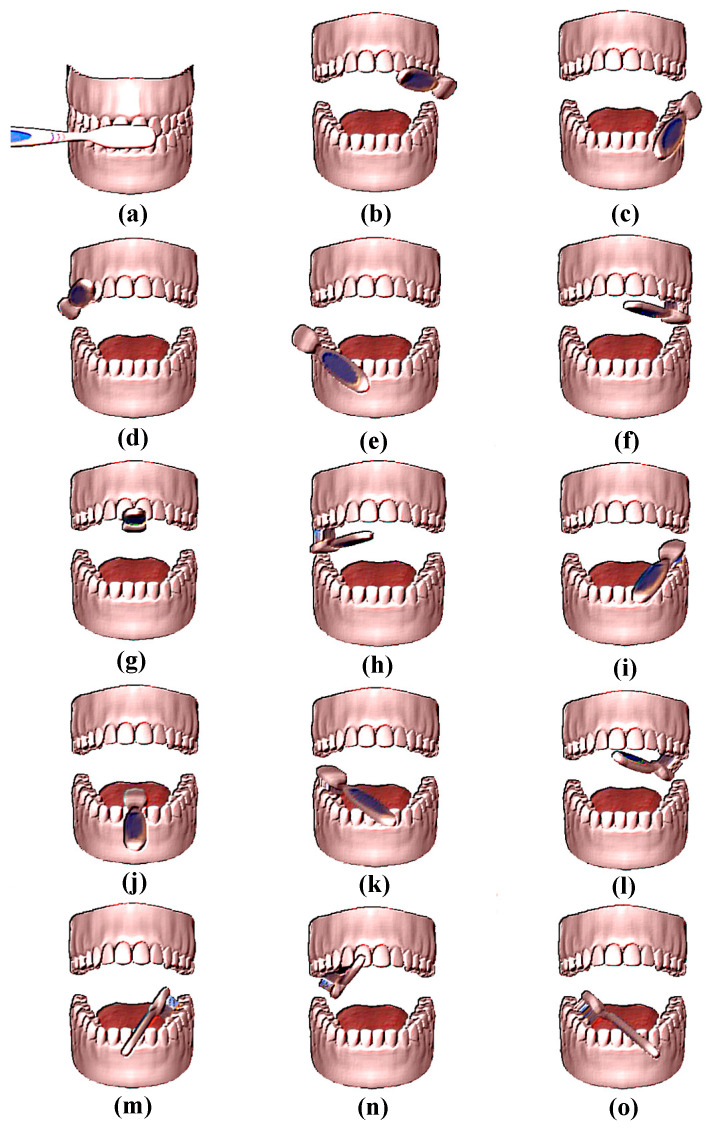
Brushing area and brushing postures. According to the Bass Brushing Technique, the brushing area proceeds from (**a**–**o**): outer incisors; upper right outer; lower right outer; upper left outer; lower left outer; upper right flank; upper incisor; upper left flank; lower right flank; lower incisor; lower left flank; upper right inner; lower right inner; upper left inner; lower left inner.

The attitude angle can indicate the direction of motion of the object in the three-dimensional space. When the teeth of different regions are brushed, the attitude angle will also change, and so, the attitude angle can be used as the distinguishing feature of the brushing area. Figure 3 illustrates the posture of the toothbrush when brushing different areas with the Bass Brushing Technique. This study classifies the brushing area by the attitude angle characteristics of different areas when brushing teeth.

## 3. Deep Learning for Brushing Posture Recognition

In the feedforward neural network, data are transmitted in one feedforward direction. Each input can be regarded as independent to the preceding input, and so, the network output only depends on the current input data. However, in the real world, the time series sensor data are generally streaming and time-dependent. The characteristics of such data depend on time, and thus, the input of the neural network is not only related to the input of the current moment but, also, related to the past time period. The input is related to the output.

### 3.1. Deep Neural Network Architecture and Learning Algorithms

In order to design a neural network for brushing gesture recognition, we first explored three mainstream deep neural networks: Convolutional Neural Networks (CNN), recurrent neural networks (RNN), and Long- and Short-Term Memory Models (LSTM). We will use these deep learning network architectures to design innovative neural networks for smart toothbrushes.

#### 3.1.1. Convolutional Neural Network

The Convolutional Neural Network (CNN) is the most well-known neural network model in deep learning. It has excellent performance in image recognition and is widely used for identification.

The CNN neural network architecture is mainly composed of a Convolutional Layer, a Pooling Layer, and a Fully Connected Layer. The convolutional layer is used to filter the original image or is called a kernel (convolution) to extract the features of the image. The pooling layer usually uses the Max Pooling method, which is mainly used to reduce the size of the feature matrix, cut down the number of calculation parameters, avoid over-fitting of the neural network (overfitting), and retain important feature information. Part of the fully connected layer is used to classify and output the features extracted by the previous layers of the network.

#### 3.1.2. Recurrent Neural Network

**Figure 4:**
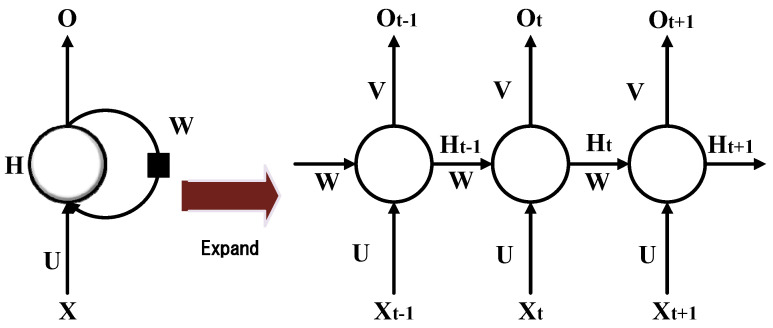
Recurrent neural network.

A recurrent neural network (RNN) is a kind of deep neural network that recurrently transmits information in its own neural network and accepts the input of time series data structure. Hence, it can be used to describe the behavior of dynamic time. Its architecture is shown in Figure 4.

In Figure 4, X is the input data at a certain moment; O is the output data at a certain moment; H is the hidden state; *U* is the weight from the input layer to the hidden layer, and the original input is abstracted and sent to the hidden layer; *V* is the weight from the hidden layer to the output layer, further abstracting the representation learned from the hidden layer; *W* is the weight from hidden layer to hidden layer, responsible for controlling and scheduling the memory of the network and $X_{t}$ represents the input at time *t*. The data are returned to the neural network operation. The general flow is $X_{t}$, and the parameters *U*, *W* and $H_{t-1}$ are calculated to produce $H_{t}$, which will be stored in the memory of the neuron, and $H_{t}$ and the parameter V will be calculated to produce $O_{t}$.

The recurrent neural network has a feedback mechanism. The hidden layer H is connected with the hidden layer H at the previous moment. At time t, the value of $H_{t}$ is the hidden layer value $H_{t-1}$ of the previous moment and the input value $X_{t}$ of the current moment. The function of the composition, F(x), is the excitation function of the hidden layer.

The right side of Figure 4 is the unfolding part of the hidden layer H, $O_{0}L,\;O_{t}\;$is the label sequence, $X_{0}L,\;\;X_{t}$ is the input sequence, $O_{t}$ is estimated by compressing all historical information in the past in H, and the parameters of the recurrent neural network are in the sequence data time. Sharing parameters can make the model less complex and present better promotion.

#### 3.1.3. Long Short-Term Memory Model

**Figure 5:**
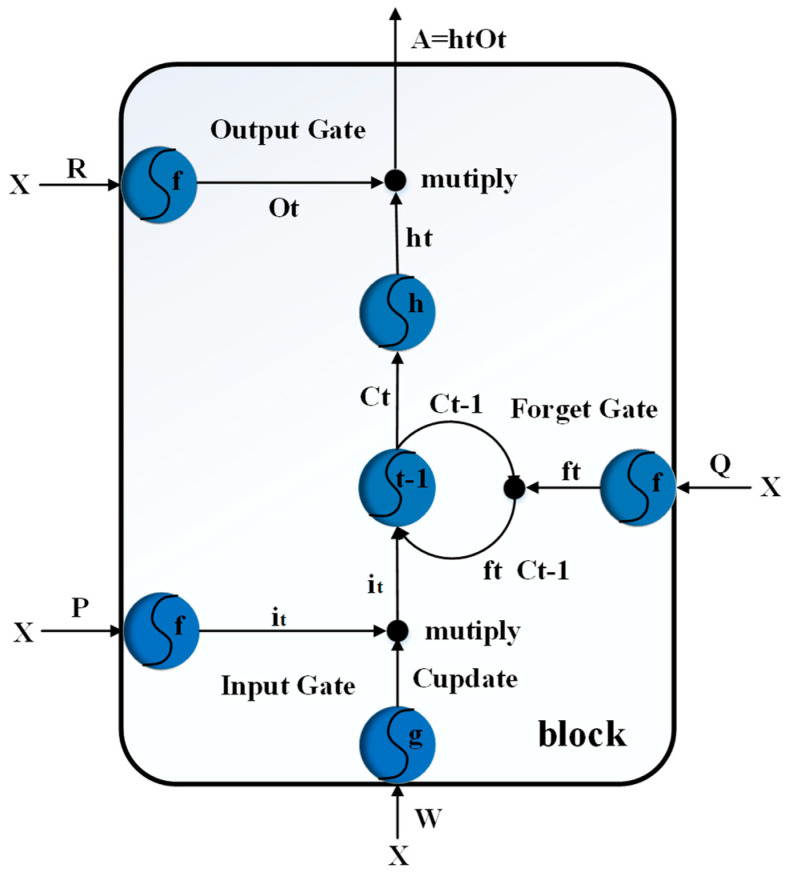
Long- Short-Term Memory (LSTM) block.

In 1997 the recurrent neural network containing Long Short-Term Memory (LSTM) [41,42,43,44,45] was proposed by German researchers Sepp Hochreiter and Juergen Schmidhuber. The road evolved and was improved and promoted by Alex Graves in recent years. Although it is theoretically possible to retransmit the neural network to the long-term correlation of data sequences, the recurrent neural network (RNN) is prone to the problem of gradient disappearance. When the gradient disappears, the neural network only learns the short-term dependence of the data sequence—that is, when the number of layers of the neural network becomes greater, the deeper the hidden layer nodes are and the shallower the hidden layer nodes. With the perceived ability declining, LSTM adds a core element memory cell (Cell) to solve the problem of the disappearance of the recurrent neural network (RNN) gradient. Each memory in LSTM contains an input gate, output gate, forget gate, and LSTM block unit, as shown in Figure 5.

LSTM uses memory to strengthen the current decision and uses three control gates to determine the storage and use of memory. In addition to the predicted output, a memory branch is added, which is updated over time, and the current memory is represented by the Gt symbol. “Forget Gate” and “Input Gate” decide whether to update the memory.

Input Gate:

Input Gate (indicated by $i_{t}$): determines whether the current input and the newly generated memory cell are added to the long-term memory. Data can be written to memory cells when the input gate is turned on via a sigmoid transfer function:

(3) $$i_{t}=Sigmoid(PX)$$

Multiply $i_{t}$ and $C_{update}$ to get $i_{t}\;C_{update}$.

If $i_{t}=0$, then $C_{update}$ means that the memory cells cannot be written; $i_{t}=1$ means $C_{update}$ cannot write to the memory cells.

Output Gate:

Output Gate: determines whether the current word sentence is added to the output. This valve is also a Sigmoid function, indicating whether to add it or not and decide whether to read out the values in the memory cells, as in Formula (4):

(4) $$O_{t}=Sigmoid(RX)$$

Multiply *A* and *B* to get:

(5) $$A=h_{t}O_{t}$$

If $O_{t}=0$, then B cannot be read through the Output Gate; $O_{t}=1\;$means that $h_{t}$ can be read through the Output Gate.

Forget Gate:

The Forget Gate is denoted by f. If the current sentence is a new topic or the opposite of the previous sentence, then the previous sentence will be filtered out by this valve; otherwise, it may continue to be retained in memory. Determine when you want to forget the cell memory:

(6) $$f_{t}=Sigmoid(QX)C_{t}$$

$f_{t}$ and $C_{t-1}\;\mathrm{are}\;$multiplied to get $f_{t\;}C_{t-1}$.

For whether the long-term memory is added to the output (Output), the tanh function is usually used. The value falls between [–1, 1], and the −1 table removes the long-term memory. If $f_{t}=0$, then the Forget Gate is closed, and $C_{t-1}$ with the previous memory unit will be deleted; if $f_{t}=1$, then the Forget Gate is enabled, and $C_{t-1}$ with the previous memory unit will be deleted.

Memory cell update:

(7) $$C_{t}=f_{t}C_{t-1}+i_{t}C_{update}$$

$C_{t\;}$ is the value of the most recent memory cell, and H(x) usually uses the Activation Function.

This study combines the nine-axis inertial sensing signal with the Euler angle eigenvalue as the input of the CNN and LSTM neural network. The weight of the CNN and LSTM neural network is then trained, and the long time series data of the LSTM network is established through the LSTM cyclic memory unit. This trained model can be used to predict the posture and position of brushing.

## 4. Recurrent Probabilistic Neural Network

With references to the concept and formalism of recurrent neural networks (RNN) and the Long-Term and Short-Term Memory (LSTM) model, when combined with the inference mechanism of a probabilistic neural network, we designed a recurrent probabilistic neural network suitable for toothbrush attitude recognition to support the Bass brushing technique.

We borrowed the long-term and short-term memory cell concept of RNN and LSTM, so that the signal input to the probabilistic neural network (PNN) can be feedback and remembered in the network. This allows the recurrent probabilistic neural network (RPNN) to correctly identify the time-dependent continuous brushing posture.

In 1988, DF Specht proposed the probabilistic neural network (PNN) [46]. PNN is a four-layer neural network architecture. Probabilistic neural networks are widely used, such as for object tracking and imaging. There are also related applications in the field of processing [47,48,49]. PNN belongs to the feedforward neural network architecture. The main theoretical basis is based on Bayesian classifiers.

**Figure 6:**
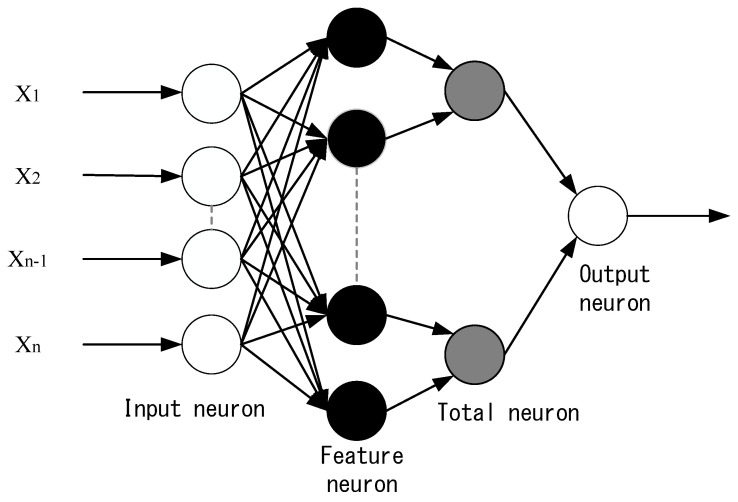
Probabilistic neural network architecture.

The probabilistic neural network architecture is shown in Figure 6, which is the input layer, the hidden layer, the sum layer, and the output layer. The feature vector $X=\left\{X_{1},X_{2},\ldots \ldots ,X_{N}\right\}$ of the probability-based neural network input layer can be any custom feature for classification, and the hidden layer is marked. The characteristic data, which are the characteristics of the input classification, are used to count the probability values of each classification through the summation unit. The summation unit corresponds to the output classification of the output layer. The number of output neurons of the output layer is the same as the number of classifications, and the output of the output layer is calculated by the summation unit. Therefore, the possibility of being classified is the highest.

The advantage of the probabilistic neural network is that its input vector size and type are not limited. It can be widely used in different types of problems. When the system is facing environmental changes and needs to add new training materials, only the newly marked training is needed. Data are added to the network or added to the corresponding new classification weights. There is no need to change the overall network architecture like other types of neural network architectures, and the weights are retrained through an iterative process. Therefore, network learning is very fast and suitable for use in real-time systems.

We use the probabilistic neural network as the core model of the neural network to identify the gesture of brushing. Combined with the RNN and LSTM models, we form a recurring probabilistic neural network, which allows the probabilistic neural network to have long-term and short-term memory functions. A continuous brushing motion sensing signal is taken from the toothbrush, and the recurrent probabilistic neural network continuously recognizes the brushing area by the motion attitude angle.

**Figure 7:**
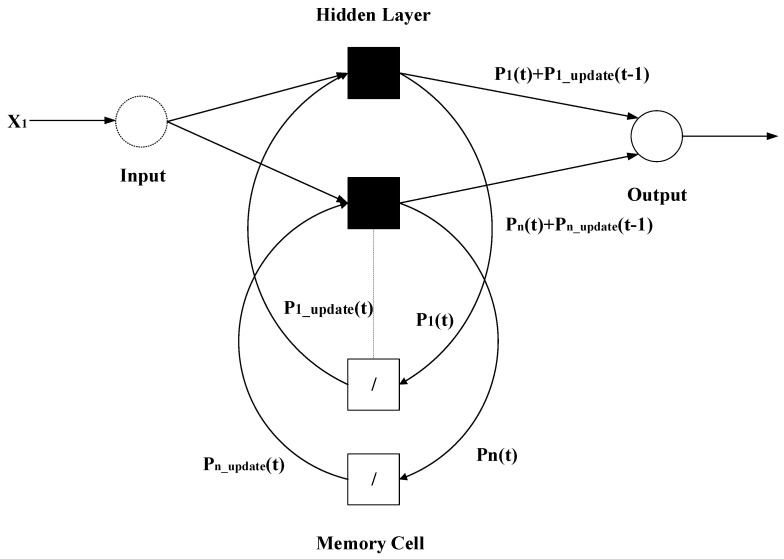
Recurrent-probabilistic-neural-network-model.

The recurrent probabilistic neural network we proposed is shown in Figure 7. The white square is the memory neuron. The output probability value is output as the neural network weight through the excitation function in the memory neuron. The memory unit updates the equation, such as:

(8) $$\begin{array}{l} P_{i\_update}(t)=(1-\delta )\cdot (P_{i\_update}(t-1)+P_{i}^{*}(t)),\;if\;P_{i}(t)\;=\;\begin{array}{c} \max\\ i \end{array}P(t)\\ P_{i\_update}(t)=\delta \cdot (P_{i\_update}(t-1)+P_{i}^{*}(t)),otherwise \end{array}$$

and

(9) $$P_{i}^{*}(t)=\left\{ \begin{array}{l} K\cdot P_{i}(t),ift\neq 0\\ 0,otherwise \end{array}\right.$$

where δ is the forgetting factor of the memory unit, K is a parameter of the length of the memory, P(t) is the probability of dividing into the time, and $P_{i_{update}}\left(t\right)\;$s the output value after the memory cell is updated.

The parameter δ in the probabilistic neural network is the Gaussian function smoothing coefficient. Each training sample can be regarded as a Gaussian function in the multidimensional feature space. The smoothness coefficient δ determines the breadth of its distribution. The larger the $\delta^{2}$ value is, the wider the distribution is, and the higher the noise is that can be tolerated. The smaller the $\delta^{2}$ value is, the narrower the distribution, the lower is the noise that can be tolerated, and δ is classified according to different classifications.

In order to make the neural network adaptive, we used the particle swarm optimization (PSO) algorithm to adjust the  δ parameters and the δ and K parameters in the recurrent neural unit to model the accuracy as a particle. The fitness function of the group optimization algorithm iterates the particles in the search space to the optimal solution in order to obtain a robust recognition performance.

Model migration.

**Figure 8:**
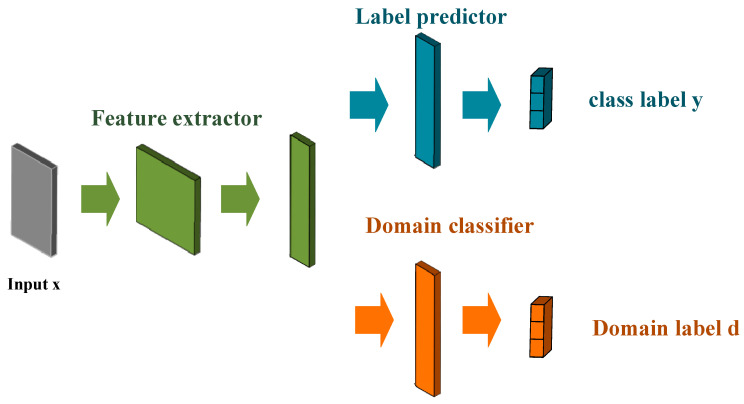
Brushing gesture recognition model migration.

The probabilistic neural network does not require a complicated training process to adjust the overall neural network architecture. However, when using the probabilistic neural network for identification, in order to improve the accuracy of model identification, the model is often complicated and large, so that the identification consumes a large amount of memory, resources, and computing time. Therefore, we used model migration to define the data directly related to the target task, called the Target Data. The data that were not directly related to the target task were called the Source Data, and the source data in the pretraining model belonged to the mark. The data $X^{s},Y^{s}$, and the user’s brushing posture feature belonged to the unmarked target data $X^{t}$. Since each user’s brushing posture is closely related to the user’s own brushing habits, each user’s brushing method may be slightly different. We sourced the identification model of the data domain that was transformed into the identification model of the target domain, as shown in Figure 8. It effectively reduced the complexity and calculation time of the model and improved the identification accuracy.

## 5. Evaluation of Hardware/Software Integrated Smart Toothbrush

**Figure 9:**
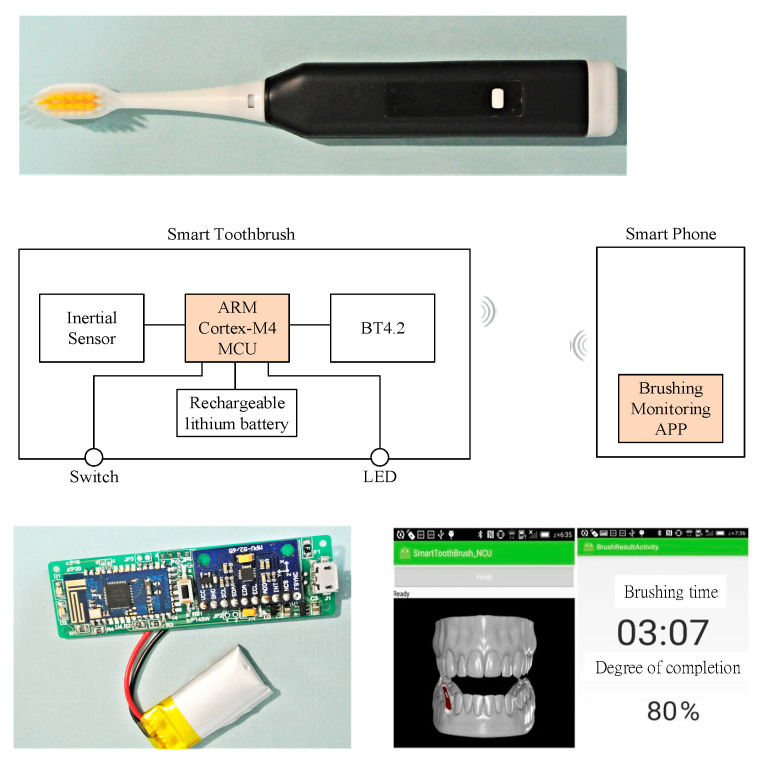
Smart toothbrush prototype system.

We developed a smart toothbrush prototype system (Figure 9) to verify the identification performance of the proposed RPNN model. A nine-axis inertial sensor MPU-9255 and Bluetooth 4.2 module were integrated into the toothbrush. The inertial sensor was used to capture the brushing motion signal, and the Bluetooth signal was transmitted to the mobile phone instantly. On the mobile phone end, we designed software to implement RPNN-based Brushing Posture Recognition and included a graphical user interface for monitoring the integrity of Bayesian brushing.

We set up brushing data for at least 15 brushing people. The toothbrush holder was 30 to 80 cm away from the subject. Each subject was sitting in front of the toothbrush holder. After watching the shell brushing method, the inertial sensing was performed. After the calibration was completed, the subject picked up the toothbrush from the toothbrush holder and brushed the teeth at the start of the prescribed brushing sequence. When brushing the teeth, the nine-axis data continuously input were filtered by the Kalman filter, and then, the data were processed by the quaternion algorithm. The fusion produced the Euler angle and, finally, extracted 5000 strokes of continuous brushing Euler angles in 15 regions. It collected five data archives in each region.

This database was used to train and test the recurrent probabilistic neural network proposed in this project to evaluate the performance and efficiency of brush gesture recognition. Next, we defined the performance evaluation indicators for brushing posture recognition in machine learning (ML), information retrieval (IR), accuracy, precision, recall, and F1 (F1-Measure). It is widely used to evaluate the pros and cons of different algorithms and models. Before understanding the above evaluation methods, it was necessary to define true positive (TP), true negative (TN), false positive (FP), and false negative (FN) as the four classifications of the dichotomy, as shown in Table 2.

- Accuracy:

It represents the ratio of the number of samples that the classifier model can correctly classify to the total number of samples for a given test dataset.

(10) $$accuracy=\frac{TP+TN}{TP+FP+FN+TN}$$

- Precision:

It represents the proportion of information that is correctly retrieved as a percentage of the material that is actually retrieved.

(11) $$precision=\frac{TP}{TP+FP}$$

- Recall:

It represents the proportion of information that is correctly retrieved as a percentage of the material that should actually be retrieved.

(12) $$recall=\frac{TP}{TP+FN}$$

- F1-Measure:

In some cases, the Precision and Recall values are contradictory. Therefore, in order to comprehensively evaluate the Precision and Recall, the most widely used method in the field of machine learning is the F-Measure. This method is a weighted average of Precision and Recall and is also known as the F-Score.

(13) $$F=\frac{(a^{2}+1)precision\cdot recall}{a^{2}(precision+recall)}$$

The most common F1-Measure is when a value is 1, calculated as follows:

(14) $$F1=\frac{2\cdot precision\cdot recall}{precision+recall}$$

Many studies have pointed out that, due to insufficient brushing time, the chance of dental caries and oral diseases is higher than that between brushing teeth. Most people use insufficient brushing time compared to Beller’s brushing method, which requires at least two minutes.

The plan will also assess the integrity of the brushing of each subject. The formula for assessing the integrity of the brushing is as follows:

(15) $$\mathrm{Brushing}\;\mathrm{completion}=\frac{User\;brushing\;time\;in\;the\;oral\;area}{Dentist\;stipulates\;brushing\;time\;in\;the\;oral\;area}\times 100\%$$

The experimental results will be compared to a typical CNN, recurrent neural networks, and LSTM identification results. We anticipate that the identification accuracy will go beyond the existing deep learning methods described above, while the computing time and hardware resource usage will be much lower than these existing methods.

## 6. Experiments

Experimental data were collected from five testers (user #1 to user #5). The brushing data were collected several times. Each brushing area collected 2000 training materials. The total training data totaled 150,000 pens, and there were 1000 more tests in each brushing area data.

We conducted comparative experiments of brushing posture recognition with a Convolutional Neural Network (CNN), Recurrent Neural Network (RNN), and posed recurrent probabilistic neural network (RPNN). In order to process the time series data, we used Multi-Scale Convolutional Neural Networks (MCNN) [50] as the CNN model, and LSTM [51], proposed by Yuwen Chen et al., as the recurrent neural network model. To compare the three deep learning models, all identification experiments were performed on the same PC (Intel Core i7-6770 CPU @ 3.40 GHz with 16.00 GB DDR3 2133 MHz).

The MCNN architecture consisted of four layers, encompassing two layers of convolutional layers and two layers of fully connected layers. The hyperparameters were set as shown in Table 3. The LSTM hyperparameter settings are shown in Table 4.

The DPNN model was optimized using PSO. We used PSO parameters with the inertia weight (W) initial value, social parameter (C1), cognitive coefficient (C2), number of particles, and iteration, such as in Table 5.

The experimental results are shown in Table 6 and Table 7.

The recognition accuracy of the RPNN model can reach 99.08%, and the average recognition rate is 16.2% higher than that of the CNN model. It is also 21.21% higher than the LSTM model, which greatly improves the recognition of the brushing area.

Typical deep neural network models such as the CNN and LSTM network architectures are complex, have many parameters, and are computationally intensive, requiring the use of better performing processors. The RPNN model is only one-thousandth and one-fiftieth the size of CNN and LSTM. The model parameters are also much lower than the CNN and LSTM, which can greatly save memory usage. Moreover, the real-time parameter is satisfied due to the lower computational load. Under the demand of brushing posture recognition, RPNN is more suitable for implementing edge devices with less hardware resources.

## 7. Conclusions

The existing smart toothbrush has insufficient accuracy and stability of posture recognition, and it is difficult to provide the user with correct information on the correctness and completeness of brushing. Therefore, this paper proposes a brushing attitude recognition model based on deep learning, which is applied to a smart toothbrush and can support the monitoring of the Bass Brushing Technique. Based on the brushing motion criterion of the Bass Brushing Technique, this study divided the teeth into 15 brushing areas and then defined the attitude angle of the toothbrush turning and brushing as the characteristics of the brushing area identification.

We used three deep neural networks for brushing gesture recognition in this study: Convolutional Neural Network (CNN) model MCNN for the time series data, the recurrent neural network with Long-Term and Short-Term Memory (RNN) Model LSTM, and the recurrent probabilistic neural network RPNN. The RPNN model used the probabilistic neural network as the core model of the neural network to identify the brush gesture. Referring to the long-term and short-term memory functions of the LSTM model, the probabilistic neural network has the ability to recognize continuous brushing motions and to recognize the conspicuous brushing areas.

Since each user’s brushing posture is closely related to the user’s own brushing inertia, employing a fixed identification model will make it difficult for users to deal with different brushing habits. In order to improve the user’s personalized brushing posture recognition, we propose a RPNN model migration method. The identification model of the source data domain (generalized model) is transformed into the identification model of the personalized domain, and the user model is trained by the PSO algorithm to make the model more suitable for personalized brushing habits, which can effectively improve identification accuracy. It also reduces the complexity of the model and computation time, enabling low-cost edge devices.

Compared with the deep learning neural network models CNN and LSTM, the results show that the recognition accuracy of the RPNN model can reach 99.08%, in which the average recognition rate is 16.2% higher than that of the CNN model and 21.21% higher than the LSTM model. The method proposed by this study can be adapted to implement edge devices with low hardware resources, such as smartphones lacking AI accelerators for instant brushing gesture recognition, while providing higher discrimination accuracy to ensure proper brushing during monitoring.

## Figures and Tables

**Table 1:** Range of toothbrush steering characteristic values.

Brush face-up	az>0.8 or 45>roll>−45
Brush face-down	az<−0.8 or 180>roll>150
Brush faced to the left	ax>0.8 or−150<pitch<−45
Brush faced to the right	ax<−0.8 or 45<pitch<150

**Table 2:** Binary problem definition. TP: true positive, FP: false positive, FN: false negative, and TN: true negative.

	Relevant	Nonrelevant
Retrieved	TP	FP
Not Retrieved	FN	TN

**Table 3:** Multi-Scale Convolutional Neural Networks (MCNN) hyperparameter settings.

Dropout	Learning Rate	Iterations	Batch Size
1.5	0.00100	20,000	64

**Table 4:** Long Short-Term Memory (LSTM) hyperparameter settings.

Hidden Layer	Learning Rate	Lambda Loss	Iterations	Batch Size
45	0.0015	0.0015	12,000,000	8500

**Table 5:** Particle swarm optimization (PSO) parameter settings.

Parameter Item	W	C1	C2	Particle Dimension	Number of Particles	Number of Iterations
Initial value	0.30	2.0	2.0	3	50	100

**Table 6:** Performance evaluation of brushing posture recognition. RPNN: recurrent probabilistic neural network.

User	Model	Testing Accuracy (%)	Precision (%)	Recall (%)	f1_Score (%)
#1	CNN	91.3933	94.5191	97.9214	96.19017
	LSTM	84.3933	78.7769	84.3933	81.5851
	RPNN	98.0067	98.1120	98.0100	98.0593
#2	CNN	80.9000	81.4349	80.9	81.1666
	LSTM	74.7200	76.2770	74.72	75.4985
	RPNN	99.9400	99.9400	99.9400	99.9400
#3	CNN	79.5400	78.5218	79.5400	79.0276
	LSTM	75.2400	76.3375	75.2400	75.7888
	RPNN	99.3067	99.3376	99.3067	99.3221
#4	CNN	81.6533	84.6613	87.4857	86.0504
	LSTM	70.9600	73.4142	70.9600	72.1871
	RPNN	99.5200	99.5347	99.5200	99.5273
#5	CNN	80.2133	83.4549	85.9429	81.7891
	LSTM	84.0533	83.0656	84.0533	83.5565
	RPNN	98.6200	98.7770	98.6200	98.6984

**Table 7:** Comparison of the computing resources of the three deep learning models.

	Model Size	Number of Parameters	Average Accuracy
CNN	285 KB	3371	82.88
LSTM	560 KB	33,630	77.87
RPNN	5 KB	453	99.08

## Data Availability

The data presented in this study are available on request from the corresponding author. The data are not publicly available due to confidentiality agreement with the company.
